# A novel pooled milk test strategy for the herd level diagnosis of *Dictyocaulus viviparus*

**DOI:** 10.1016/j.vpoa.2019.100008

**Published:** 2019-04-07

**Authors:** Catherine McCarthy, Johan Höglund, Rob Christley, Mikael Juremalm, Inna Kozlova, Robert Smith, Jan van Dijk

**Affiliations:** a University of Liverpool, Department of Epidemiology and Population Medicine, Institute of Infection and Global Health, Liverpool, CH64 7TE, UK; b Swedish University of Agricultural Sciences, Department of Biomedical Sciences and Veterinary Public Health, Section for Parasitology, P.O. Box 7036, Uppsala, Sweden; c Boehringer Ingelheim Svanova, P.O. Box 1545, SE-751 45, Uppsala, Sweden; d University of Liverpool, Department of Livestock Health and Welfare, Institute of Veterinary Science, Liverpool, CH64 7TE, UK; e Animal Health Trust, Epidemiology and Surveillance, Centre for Preventive Medicine, Lanwades Park, Kentford, Newmarket, Suffolk, CB8 7UU, UK

**Keywords:** *Dictyocaulus viviparus*, Dictyocaulosis, Diagnosis, ELISA, Dairy cow, Milk, ROC analysis

## Abstract

Current diagnostic methods for detecting the presence or absence of *Dictyocaulus viviparus* in dairy herds, are insensitive when based on detection of antibody levels in bulk tank milk (BTM). Here we present a novel technique to confirm the presence of the parasite based on a pooled-milk sample from 10 randomly selected first – lactation heifers (FLH). This study was run in two parts. First, a longitudinal study was performed to look at infection dynamics in milk samples across the grazing season using a prototype ELISA developed by Svanova (Boehringer-Ingelheim, Uppsala). We identified that mean ODR values in milk samples from FLH was significantly higher than that for older cows (0.13 versus 0.07 respectively, p < 0.001) suggesting that samples from the FLH cohort should be pooled to produce the test. Second, the pooled – milk test was evaluated on a cross-sectional survey of UK dairy herds (n = 25 grazing and n = 25 zero-grazing herds) to evaluate test performance under field conditions. The optical density ratio (ODR) cut-off value for our pooled-milk test using 10 FLH milk samples was optimal at a value of 0.16. Pooling 10 FLH samples created a sensitivity and specificity of 66.7% and 95.5% respectively. In comparison, whole-herd BTM samples had a maximum sensitivity of 37.5% and specificity of 63.6% at an ODR cut-off of 0.18. The area under the curve according to receiver-operative-characteristic (ROC) analysis was high for the 10-heifer test (0.87) but poor for the whole herd BTM testing (0.45). This study provides a more sensitive diagnostic test strategy for the screening of *D.viviparus* in dairy herds. Testing herds at the end of a grazing season would facilitate the planning of effective control measures, such as the use of the lungworm vaccination or strategic deworming, for the following grazing season. This may prove to be a useful test strategy for the diagnosis of a variety of parasitic diseases of livestock.

## Highlights

• First-lactation heifers have higher milk antibody levels than older animals.
• The optimum test is produced when 10 first-lactation heifer samples are pooled.
• The pooled test is superior to the bulk tank in diagnosing lungworm in a herd.

## 1 Introduction

The parasitic disease dictyocaulosis, or ‘husk’, caused by the bovine lungworm, *Dictyocaulus viviparus*, is a leading cause of morbidity and mortality in grazing dairy cattle in temperate regions (David, 1999). Milk production losses due to husk have been estimated at 4 kg/cow/day for clinical outbreaks (Holzhauer et al., 2011) with subclinical infections carrying an average estimated loss of 0.5 kg/cow/day (Charlier et al., 2016). Using insensitive bulk tank milk (BTM) tests, dairy herd prevalence levels were shown to vary between 2.9% in Switzerland (Frey et al., 2018), 9% in conventional herds and 18% in organic herds in Sweden (Höglund et al., 2010), 21.1% in Germany (Klewer et al., 2012) and 62.8% in Ireland (Bloemhoff et al., 2015). Robust estimates are lacking for UK grazing herds but the incidence of outbreaks of dictyocaulosis has increased dramatically since the second half of the Nineties (McLeonard and Van Dijk, 2017).

Within adult dairy herds, transmission of *D.viviparus* may occur without obvious clinical signs. Knowing the ‘lungworm status’ of a farm is pertinent to the design of appropriate and sustainable parasite control strategies. In herds where *D.viviparus* is absent, control measures should be targeted towards biosecurity measures. In herds where *D.viviparus* is circulating, control measures should target the presence and maintenance of herd – level immunity. An ELISA based on a recombinant major sperm protein (MSP) was first developed by von Holtum et al. (2008) and then adapted for use in milk samples by Fiedor et al. (2009). Initial studies indicated that the BTM test had a favourable sensitivity and specificity (100% and 97.3% respectively) when within-herd seroprevalence levels exceeded 20% (Schunn et al., 2012). However, later studies did not support these findings, with lower sensitivity and specificity values of 83% and 95% reported, which further decreased to 56% and 93% when the within-herd prevalence level decreased to below 10% (Ploeger et al., 2014). In re-infected animals there is normally a short window of seropositivity, making it difficult to diagnose the presence of lungworm in adult dairy herds (Strube et al., 2017). Cows which have been exposed previously may not show any significant increase in antibody levels when re-exposed to the parasite (Strube et al., 2017). BTM samples are therefore likely to be false negative for *D. viviparus*, especially outside of peak windows of parasite transmission.

Testing individual animals has been advocated to identify *D. viviparus* within dairy herds. In order to achieve a 95% confidence in diagnosing the parasite in herds coughing at grass in the Netherlands, it was shown that serology from six randomly selected first – lactation heifers (FLH) should be tested (Ploeger et al., 2012). Whether this applies to non-clinically affected herds is unknown. Furthermore, this is likely to be an expensive testing regime, making it unfeasible for routine herd monitoring. A milk based test could be expected to be more widely adopted by the dairy industry.

Our aim was to develop a novel test strategy which would have a superior sensitivity to the current bulk tank test. We hypothesised that pooling milk from a subset of FLH is likely to target the animals with the highest antibody levels in the milking herd. Testing a subset of the herd could prevent the dilution effect seen in the bulk tank and so may be able to detect herds with a lower within – herd prevalence level than BTM testing. First, we performed a longitudinal study on individual animals to determine how many milk samples should be tested. Pooled milk samples from FLH were then tested and subsequently validated in a cross-sectional study of UK dairy herds.

## 2 Methods

This study was reviewed and approved by the University of Liverpool Veterinary Research Ethics committee (VREC431).

### 2.1 Study 1: Longitudinal study design and selection of farms

Farms from Great Britain were recruited for the initial longitudinal study. Inclusion criteria for participation consisted a minimum herd size of 100 cows, a veterinary diagnosis (through detection of larvae in faeces, raised serum or milk antibody levels or post-mortem findings) of *Dictyocaulus viviparus* within the past 5 years, and lactating cattle grazing pasture during the summer months (at least from July to September). Inclusion criteria further included monthly milk sampling provided by the National Milk Records (NMR) services.

Data from a preliminary longitudinal trial where 60 individual animals were tested on 5 different farms twice a year for two consecutive years was used to perform a sample size calculation [unpublished data]. The minimum number of herds to be sampled to be more than 95% sure to detect within year differences in antibody levels was four (see supplementary information).

A cohort of 40 FLH were randomly selected from each farm using a computerised random number generator. If fewer than 40 FLH were milked on any occasion, then all available FLH were included. Animals who missed a month’s milk recording through cessation of lactation, illness or removal from the herd were replaced by another randomly selected FLH. Individual milk samples from the FLH cohort on each farm were collected at monthly intervals during August, September and October 2016, plus once in May 2017.

### 2.2 Designing a pooled-milk test

The pooled-milk test, ${\;P}_{n}$, would be based on mixing a 1 ml milk sample from n number of FLH. The number of samples n could be between 1 and 20. An upper limit of 20 was set as it was considered that pooling more than 20 samples would prove too impractical for routine testing. It was also a prior assumption that $P_{10}$ would be both practical and easy to facilitate for a commercial test.

For initial test development, the effect of creating and testing $P_{n\;}\text{w}\text{h}\text{e}\text{r}\text{e}\;1\leq n\leq 20$ was simulated by bootstrap sampling, with replacement, the ODR results from  n individuals in each herd and month in the longitudinal study and calculating the means from individual ODRs. The bootstrap process was repeated 1000 times to calculate the median ODR and 95% confidence limits (CLs). The widths of the CLs in each herd, month and value of  n was taken as separate data points. The Kruskal Wallis and the Mann-Whitney U post-hoc test were used to assess the significance between different pairs of n. The minimum number of samples which did not significantly widen the confidence intervals from $P_{20}$ was noted as $\;P_{s}$.

### 2.3 Milk sample processing and testing

Milk samples were defatted by centrifuging at 2000 x g for 15 min before separating the supernatant from the lipid layer. Individual milk samples had been pre-treated with the preservative bronopol, according to NMR’s policy. Defatted samples were stored at −20 °C until tested.

All samples were tested using a prototype of an ELISA plate developed by Svanova (Boehringer-Ingelheim Svanova, Uppsala). The ELISA procedure is a solid phase indirect ELISA. Plates (Polysorp, Nunc) were coated in *D. viviparus* non-infectious major sperm protein (MSP) antigen and incubated overnight at room temperature with some modifications as described in Goździk et al. (2012). Positive and negative serum controls were diluted 1:100 in PBS containing 0.05% Tween-20 (PBS-Tween). Plates were incubated for 1 h at room temperature with 100 μl/well pre-diluted positive and negative controls and undiluted skimmed milk. After three washes in a plate washer (Thermo Scientific, Wellwash) using PBS-Tween and tapping dry, plates were incubated with 100 μl/well horseradish peroxidase-conjugated anti-bovine IgG for 1 h at room temperature. Plates were washed as before, tapped dry and incubated with 100 μl/well tetramethylbenzidine in hydrogen peroxide solution for 10 min in the dark at room temperature. The enzymatic colour reaction was stopped by adding 50 μl/well 2 M sulphuric acid. Optical densities (OD) were then read on a microplate photometer (Thermo Scientific, Multiskan EX) within 10 min at a wavelength of 450 nm. Results were expressed as an optical density ratio (ODR) using the same formula as in the previous commercial *D.viviparus* ELISA (von Holtum et al., 2008):

$$ODR\;=\;\frac{{{OD}_{test\;specimen}-\;OD}_{blank}}{{OD}_{positive\;control}-\;{OD}_{blank}}$$

For pooled-milk testing, a 1 ml sample was pipetted from each individual skimmed milk sample whilst under constant homogenisation using an electronic stirrer. The pooled-milk sample underwent constant homogenisation for two minutes prior to testing according to the ELISA protocol.

### 2.4 Study 2 Cross-sectional study: Test performance under field conditions

To understand the performance of the pooled-milk test under field conditions, a second study, based on cross-sectional sampling of the Tesco Sustainable Dairy Group (TSDG) herds, was performed. The TSDG is a group of 600+ dairy herds in England, Scotland and Wales, of which 10% operate a closed zero-grazing system. A questionnaire was sent to all grazing herds to gain farmers consent for the study. This also allowed a binary classification of the herd into either those that had or had not had a veterinary diagnosis of lungworm within the previous 2 years (through detection of larvae in faeces, raised serum or milk antibody levels or post-mortem findings). Individual and BTM samples from grazing herds were collected once during September to November 2017 from the relevant milk recorders (National Milk Records, Quality Milk Management Services and Cattle Information Services). In addition, closed zero-grazing herds who bred their own replacement FLH, milk recorded with NMR and had milk samples available for scientific use, had their individual and BTM samples analysed once during January to March 2018. For all grazing and zero-grazing herds, $P_{s}$ and $P_{10}$ were created and tested along with the BTM sample.

To explore the effect of varying the positivity ODR cut-off value, a receiver-operating characteristic (ROC) curve analysis, as has previously been described (Schunn et al., 2012), was used to compare $P_{s}$, $\;P_{10}$ and the BTM sample ODRs. True positive herds were assumed to be grazing herds which had received a veterinary diagnosis of lungworm within the preceding 2 years, whereas true negative herds were assumed to be the closed zero-grazing herds. The area under the curve (AUC), sensitivity, specificity and likelihood ratios (LR) of the pooled-test were calculated in R using equations provided by Thrusfield (2005). The optimum cut-off was taken as that which maximised both sensitivity and specificity.

## 3 Results

### 3.1 Study 1: longitudinal sampling of individual FLH

Four farms met the inclusion criteria and were selected for the initial longitudinal study. The average number of lactating animals per herd was 231 (median 180, range 165–400); of which 69 were FLH in their first lactation (median 46, range 35–150) and 162 were cows of parity 2 and above (median 143, range 114–250). These herd sizes were representative of the average size of UK dairy herds in 2016 (AHDB Dairy, 2017). A total of 501 individual milk samples from FLH were tested from August 2016 to May 2017. It was not possible to collect one herd’s milk samples in May 2017 and so their April milk samples were tested instead. Additionally, one herd in September, October and May did not have any milk samples available for testing.

### 3.2 Calculating the optimum number of milk samples to enter the pooled-milk test

**Fig. 1:**
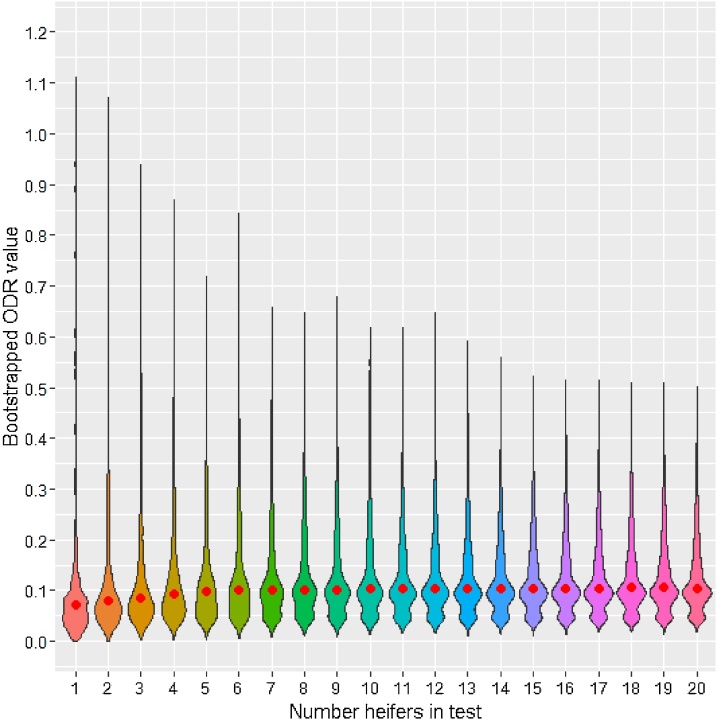
Distributions of optical density ratio (ODR) values with 95% confidence limits when between 1 and 20 heifer milk samples were pooled and tested from all months (via bootstrap analysis with 1000 iterations). Red circle refers to median ODR value when n number of heifer samples are pooled and tested.

**Fig. 2:**
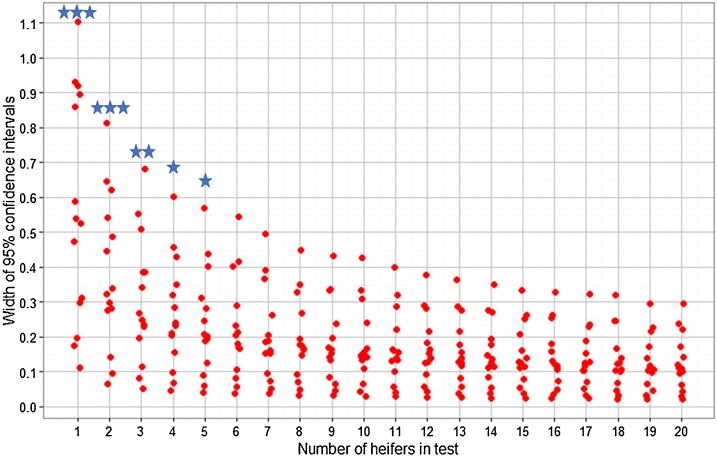
Widths of the 95% confidence limits when between 1 and 20 heifers are randomly selected to enter the pooled-milk test. Each red dot refers to the size of the confidence limits when heifers from 1 farm and 1 month were selected and bootstrap sampled 1000 times. Red dots are horizontally staggered for clarity. Blue stars relate to groups of widths which are significantly wider than selecting 20 heifers (*p ≤ 0.05, **p ≤ 0.01, ***p ≤ 0.001).

The effect of increasing the number of bootstrapped FLH samples entering the pooled-milk test ($P_{n}$) on the narrowing of ODR distributions is demonstrated in Fig. 1. Although the median bootstrapped value changed by only 0.03 when different $P_{n}$ values were used, the widths of the 95% confidence intervals (CI) were significantly related to $P_{n}$ (H_(19)_ = 54.1, p < 0.001). Across all herds and months, the narrowest CI widths occurred when $P_{20}$ whereas $P_{n\leq 5}$ created significantly broader CIs than $P_{20}$ (Fig. 2). Thus, regardless of herd size, the minimum number of FLH to be included to detect at least one animal with 95% confidence was six animals. The relative decrease in CI is small if more than 9–10 samples are included (Fig. 2). For further validation of the test, a 6-heifer test and a 10-heifer test were compared.

### 3.3 Study 2: evaluating the dynamics of the pooled-milk test in a cross-sectional study

A total of 148 grazing herds from across the UK consented to inclusion in the bulk and pooled-milk testing although four herds did not have a representative BTM and 52 herds did not provide individual cow samples for pooled-milk sampling. Furthermore, two herds did not have 10 FLH and so only the 6-heifer pooled-milk sample was available. This left 90 grazing herds where BTM, 6-FLH and 10-FLH pooled-milk samples were available. Out of the 90 herds, 25 reported that they had a veterinary diagnosis of lungworm confirmed within the preceding 24 months. Milk samples from an additional 25 zero-grazing herds were accessible for inclusion in the pooled-FLH and BTM testing.

#### 3.3.1 Determining the optimum ODR cut-off value using receiver-operating-characteristics analysis

**Fig. 3:**
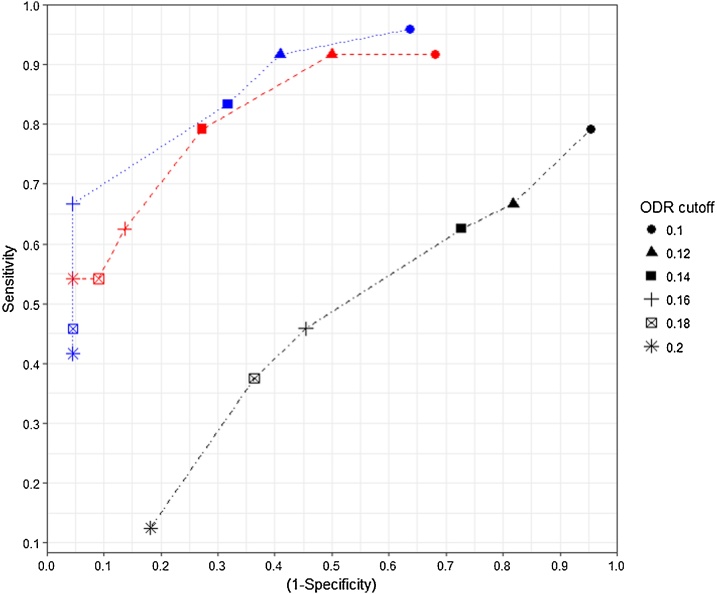
ROC curve showing results from pooled-milk and bulk tank testing. Blue (dotted line) and red (hashed line) indicate when either 10 or 6 heifer milk samples have been pooled and tested respectively (${\text{P}}_{10}$ and ${\text{P}}_{6}$). Black line shows the bulk tank results.

Results from the receiver-operating-characteristics (ROC) analysis can be seen in Fig. 3. The area under the curve (AUC) for $P_{10}$ is high at 0.87 and shows maximal sensitivity and specificity at a cut-off of 0.16 (66.7% and 95.5% respectively) (Table 1). $P_{6}$ has a lower AUC of 0.85 and has a maximal cut-off of 0.14 (sensitivity 79.2%, specificity 72.7%) (Table 2). The BTM performs less favourably than either of the pooled-FLH tests with maximal sensitivity and specificity of only 37.5% and 63.6% respectively at a cut-off of 0.18. The AUC for bulk tank testing was 0.45 and so evidently non-diagnostic in these herds (Table 3). Using a cut-off ODR of 0.16, a positive ${\text{P}}_{10}$ test result is 14.7 times more likely in a positive herd than a negative one.

**Table 1:** Sensitivity, specificity, positive likelihood ratio (LR+) and negative likelihood ratio (LR-) for the 10-first lactating heifer (FLH) test ($P_{10})$ at an optical density ratio (ODR) positivity threshold (cut-off) between 0.10 and 0.20. AUC = 0.87.

ODR Cut-off	Sensitivity (%)	Specificity (%)	LR+	LR-
0.10	95.8	36.4	1.5	0.1
0.12	91.7	59.1	2.2	0.1
0.14	83.3	68.2	2.6	0.2
0.16	66.7	95.5	14.7	0.3
0.18	45.8	95.5	10.1	0.6
0.20	41.7	95.5	9.2	0.6

**Table 2:** Sensitivity, specificity, positive likelihood ratio (LR+) and negative likelihood ratio (LR-) for the 6-first lactating heifer (FLH) test ($P_{6})$ at an optical density ratio (ODR) positivity threshold (cut-off) between 0.10 and 0.20. AUC = 0.85.

ODR Cut-off	Sensitivity	Specificity	LR+	LR-
0.10	91.7	31.8	1.3	0.3
0.12	91.7	50.0	1.8	0.2
0.14	79.2	72.7	2.9	0.3
0.16	62.5	86.4	4.6	0.4
0.18	54.2	90.9	6.0	0.5
0.20	54.2	95.5	11.9	0.5

**Table 3:** Sensitivity, specificity, positive likelihood ratio (LR+) and negative likelihood ratio (LR-) for the bulk milk test at an optical density ratio (ODR) positivity threshold (cut-off) between 0.10 and 0.20. AUC = 0.45.

ODR Cut-off	Sensitivity	Specificity	LR+	LR-
0.10	79.2	4.5	0.8	4.6
0.12	66.7	18.2	0.8	1.8
0.14	62.5	27.2	0.9	1.4
0.16	45.8	54.5	1.0	1.0
0.18	37.5	63.6	1.0	1.0
0.20	12.5	81.8	0.7	1.1

#### 3.3.2 Comparing the 10-heifer pooled-milk test to the bulk tank milk test

**Fig. 4:**
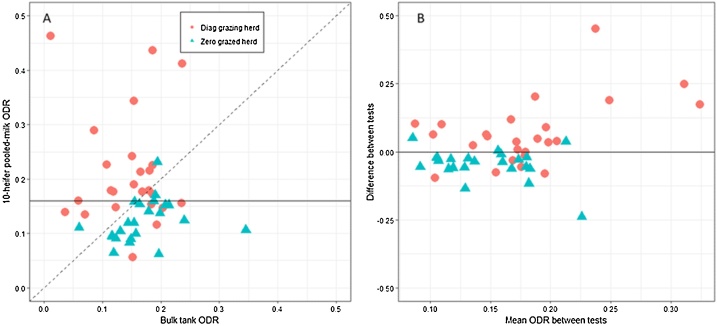
Correlation between bulk tank milk optical density ratio (ODR) and 10-heifer pooled-milk ODR in grazing herds with a veterinary diagnosis of lungworm (red circles) and zero-grazing herds (green triangle). A) shows ODR values of the two tests. The diagonal hashed line relates to line by which x = y and horizontal line the suggested positivity cut-off for the pooled-heifer test (0.16). B) Bland-Altman plot comparing difference between tests (10-heifer pooled test minus bulk tank) with mean of both tests.

In the grazing herds with a veterinary diagnosis, the 10-FLH-milk test ($P_{10}$) created higher ODR values than the BTM in 72% of herds, with mean $\;P_{10}$ ODR of 0.21 (min 0.06, median 0.18, max 0.46) compared to mean BTM ODR in the zero-grazing herds of 0.12 (min 0.06, median 0.12, max 0.23) (Fig. 4). In herds where the $P_{10}$ ODR exceeded the BTM, $P_{10}$ created an average ODR that was 0.10 higher than the BTM results (min 0.004, median 0.06, max 0.45). In samples where the BTM ODR exceeded $P_{10}$, the BTM sample was higher by an average of 0.06 (min 0.002, median 0.06, max 0.24).

## 4 Discussion

This study describes a novel adaptation of the use of easily-obtainable milk samples, using individual milk samples randomly taken from first lactation heifers (FLH) to address the low sensitivity problems associated with a bulk tank milk (BTM) test for the cattle lungworm, *Dictyocaulus viviparus*. At an ODR cut-off of 0.16, the pooled 10-FLH milk test has a sensitivity of 66.7% and specificity of 95.5%. A positive 10-FLH test result was shown to be 14.7 times more likely in a positive herd than a negative one (positive likelihood ratio). In comparison, the routinely used BTM test only provided a maximum sensitivity and specificity of 37.5% and 63.6% respectively. The pooled 10-FLH milk test functions in modern dairy herds not clinically affected by the parasite.

A first step towards controlling lungworm is to have access to diagnostics that indicate whether the parasite is present on the farm. On many farms, the current lungworm status is unknown. Knowing whether the parasite is circulating on the farm or not will enable the veterinarian and farmer to design a targeted, evidence-based, preventative medicine approach to control this potentially devastating parasite. In addition, the frequent purchase of animals has been identified as a significant risk factor for lungworm outbreaks in dairy herds in Belgium (Charlier et al., 2016). The present 10-FLH milk test can be used to assess the likely lungworm status of incoming lactating animals, thereby assessing the risk to both the main herd and the recent arrivals. Furthermore, on farms without clinical signs of dictyocaulosis, testing in the autumn would provide a useful routine test to perform at the end of a grazing season. If the results suggest that the parasite is circulating on the farm, effective control measures, such as the use of the lungworm vaccination or strategic anthelmintic treatments, could be planned for the following grazing season. Further work is needed to understand how the presence of the parasite on the farm relates to the risk of a clinical outbreak within a herd. Additionally, correlations between ODR values and production losses would be a useful tool for promoting effective lungworm control. However, this has not been established for the BTM tests and is likely to be difficult to calculate in field conditions where production losses will be heavily influenced by several farm related factors, such as grazing lengths, host genetics and production levels.

Other options for presence-absence testing rely on either a large proportion of the herd to have seroconverted, or clinical signs to have been witnessed. A previously developed bulk tank milk ELISA, for instance, appears to offer a sensitivity of 55.6% and specificity of 92.2% but only if at least 10% of the herd have seroconverted (Ploeger et al., 2014). However, if the herd needs to be tested for reasons of herd health planning, neither prior knowledge on seroconversion rates nor clinical signs will be present. In this study we demonstrated that the 10-FLH test can be used prior to clinical signs being present and in larger herd sizes with up to 400 lactating cattle.

The higher AUC for the 10-FLH-milk test (0.87) compared with the BTM test (0.45) suggests that the diagnostic value of targeted testing of first lactating heifers substantially exceeds BTM testing. The 10-FLH test can create an ODR that is 0.45 higher than in BTM whereas the antibody levels in BTM only exceeds the 10-FLH milk test by a maximum ODR of 0.24. This may suggest that the below-desirable performance of the BTM test could be due to a dilution effect of a small number of antibodies in a large volume of milk.

Randomly selecting 10 FLH is open to selection bias with farmers perhaps inclined to select animals which either have been recently coughing or showing suggestive clinical signs, such as milk drop. It is also possible that farmers and veterinarians may not sample precisely 1 ml from each animal. The effect such factors would have on the sensitivity and specificity of the test is yet to be investigated. Work on the number of animals to be included in composite faecal egg count tests has shown, however, that test outcome is sensitive to which animals are included but not to exactly how much of the sample of that animal is included (Presland et al., 2005).

Previous *D.viviparus* testing protocols advocate testing 6 individual serum samples from FLH (Ploeger et al., 2012). At current UK laboratory costs this would cost £150 (APHA Laboratory Services, 2019) plus veterinary costs associated with blood sampling. In comparison, a single pooled milk test for *Brucella abortus* costs £9.10. The test for lungworm exposure described in this paper is well-placed to become a quick, cheap, commercial test for mass use, for example performed by milk recording companies. The latter would also standardise the random sampling of heifer milk samples.

This study assumes that the grazing herds with a veterinary diagnosis represented true positive herds whereas zero-grazing herds were true negative herds. These assumptions were made in the absence of a gold - standard test. In the absence of a test with 100% specificity, true negative herds need to be implied on grounds of biological reasoning. Therefore, ‘negative’ herds which applied zero grazing and bred their own replacement FLH were included in this study. Although clinical lungworm outbreaks have occasionally been recorded in recently housed animals, the transmission of lungworm in modern permanently housed, zero grazing, herds is unlikely. True positive herds were included based on a prior veterinary diagnosis. Although participants were asked which method of diagnosis the veterinary surgeon used, it was possible that this method was unknown to the farmer, opening the possibility that this was achieved through the presence of suggestive clinical signs only. Therefore we cannot exclude the possibility that a portion of these herds may have been incorrectly identified as positive herds. However, these herds had all received a diagnosis from a veterinary surgeon, and were not classed purely on a farmer’s impression on whether they had noticed clinical signs.

This study used data collected in one year from four herds as the basis for the bootstrap analysis on how many animals to be included in the test. Thus, between-year differences in parasite burdens, as well as differences between farms, may potentially affect the results in other years. However, the test appeared to function well in 50 herds from across Great Britain without the prerequisites of a minimum within – herd prevalence level. This offers significant advantages over the current BTM testing which requires a minimum herd prevalence level of 20% (Schunn et al., 2012). Further work is needed to use the test on a larger sample size with known positive or negative status (for example, from post-mortem testing).

In conclusion, we describe a novel method of identifying the lungworm infection within dairy herds based on targeted milk sampling of first lactation heifers (FLH) which works in larger herd sizes with unknown within herd – seroconversion rates and in the absence of clinical signs. The 10-FLH test has a sensitivity of 66.7% and specificity of 95.5% and is 14.7 times more likely in a positive herd than a negative one. This represents a cheap option for presence-absence testing. This novel method may prove to be a useful test strategy in the diagnosis of various parasitic conditions of livestock.

## Funding

This work was supported the 10.13039/501100000268Biotechnology and Biological Sciences Research Council [grant number 1511062] and the University of Liverpool, UK.

## Declaration of interests

The authors declare that they have no known competing financial interests or personal relationships that could have appeared to influence the work reported in this paper.

## CRediT authorship contribution statement

**Catherine McCarthy:** Conceptualization, Methodology, Software, Formal analysis, Investigation, Writing - original draft, Project administration. **Johan Höglund:** Validation, Resources, Writing - review & editing. **Rob Christley:** Formal analysis, Writing - review & editing, Supervision. **Mikael Juremalm:** Validation, Resources. **Inna Kozlova:** Validation, Investigation, Resources. **Robert Smith:** Resources, Project administration. **Jan van Dijk:** Conceptualization, Methodology, Formal analysis, Writing - review & editing, Supervision, Project administration, Funding acquisition" does not match the list of acceptable roles. Please choose a role from the below list for this author: Conceptualization; Data curation; Formal analysis; Funding acquisition; Investigation; Methodology; Project administration; Resources; Software; Supervision; Validation; Visualization; Writing - original draft; Writing - review & editing.

## References

[bib0005] AHDB Dairy (2017). Datum UK Cow Numbers. https://dairy.ahdb.org.uk/market-information/farming-data/cow-numbers/uk-cow-numbers/.

[bib0010] Bloemhoff Y., Forbes A., Good B., Morgan E., Mulcahy G., Strube C., Sayers R. (2015). Prevalence and seasonality of bulk milk antibodies against Dictyocaulus viviparus and Ostertagia ostertagi in Irish pasture-based dairy herds. Vet. Parasitol..

[bib0015] Charlier J., Ghebretinsae A., Meyns T., Czaplicki G., Vercruysse J., Claerebout E. (2016). Antibodies against Dictyocaulus viviparus major sperm protein in bulk tank milk: association with clinical appearance, herd management and milk production. Vet. Parasitol..

[bib0020] David G. (1999). Strategies for the control of parasitic bronchitis in cattle. In Pract..

[bib0025] Fiedor C., Klewer A.-M., von Samson-Himmelstjerna G., Schnieder T., Strube C., Forbes A., Buschbaum S. (2009). Evaluation of a milk ELISA for the serodiagnosis of Dictyocaulus viviparus in dairy cows [electronic resource]. Vet. Parasitol..

[bib0030] Frey C.F., Eicher R., Raue K., Strube C., Bodmer M., Hentrich B., Gottstein B., Marreros N. (2018). Apparent prevalence of and risk factors for infection with Ostertagia ostertagi, Fasciola hepatica and Dictyocaulus viviparus in Swiss dairy herds. Vet. Parasitol..

[bib0035] Goździk K., Engström A., Höglund J. (2012). Optimization of in-house ELISA based on recombinant major sperm protein (rMSP) of Dictyocaulus viviparus for the detection of lungworm infection in cattle [electronic resource]. Res. Vet. Sci..

[bib0040] Höglund J., Dahlström F., Engström A., Hessle A., Jakubek E.-B., Schnieder T., Strube C., Sollenberg S. (2010). Antibodies to major pasture borne helminth infections in bulk-tank milk samples from organic and nearby conventional dairy herds in south-central Sweden. Vet. Parasitol..

[bib0045] Holzhauer M., van Schaik G., Saatkamp H.W., Ploeger H.W. (2011). Lungworm outbreaks in adult dairy cows: estimating economic losses and lessons to be learned. Vet. Rec..

[bib0050] Klewer A.-M., Forbes A., Schnieder T., Strube C., Schnieder T., Forbes A., Schnieder T., Strube C. (2012). Short communication: a survey on Dictyocaulus viviparus antibodies in bulk milk of dairy herds in Northern Germany. Prev. Vet. Med..

[bib0055] McLeonard C., Van Dijk J. (2017). Controlling lungworm disease (husk) in dairy cattle. In Pract..

[bib0060] Ploeger H.W., Verbeek P.C., Dekkers C.W.H., Strube C., Van Engelen E., Uiterwijk M., Lam T.J.G.M., Holzhauer M. (2012). The value of a bulk-tank milk ELISA and individual serological and faecal examination for diagnosing (sub)clinical Dictyocaulus viviparus infection in dairy cows. Vet. Parasitol..

[bib0065] Ploeger H.W., Holzhauer M., Uiterwijk M., Van Engelen E. (2014). Comparison of two serum and bulk-tank milk ELISAs for diagnosing natural (sub)clinical Dictyocaulus viviparus infection in dairy cows. Vet. Parasitol..

[bib0070] Presland S.L., Morgan E.R., Coles G.C. (2005). Counting nematode eggs in equine faecal samples. Vet. Rec..

[bib0075] Schunn A.-M., Strube C., Schnieder T., Forbes A. (2012). Validation of a Dictyocaulus viviparus MSP-ELISA and cut-off adjustment in a one-year longitudinal field study in dairy cattle herds [electronic resource]. Vet. Parasitol..

[bib0080] APHA Laboratory Services (2019), Available at: http://apha.defra.gov.uk/apha-scientific/services/lab/ (Accessed 19 November 2018).

[bib0085] Strube C., Springer A., Schunn A.M., Forbes A.B. (2017). Serological lessons from the bovine lungworm Dictyocaulus viviparus: antibody titre development is independent of the infection dose and reinfection shortens seropositivity. Vet. Parasitol..

[bib0090] Thrusfield M. (2005). Veterinary Epidemiology.

[bib0095] von Holtum C., Strube C., Schnieder T., von Samson-Himmelstjerna G. (2008). Development and evaluation of a recombinant antigen-based ELISA for serodiagnosis of cattle lungworm. Vet. Parasitol..

