# Supplementary material for: A novel pooled milk test strategy for the herd level diagnosis of Dictyocaulus viviparus
Source: Vet Parasitol X. 2019 Apr 7;1:100008. doi: 10.1016/j.vpoa.2019.100008 (PMC6788444; doi:10.1016/j.vpoa.2019.100008)
Supplement: Supplementary file 1 [file mmc1.docx]

**Supplementary Information: Sample size calculation**

**Methods**

A sample size calculation (number of cows to be sampled per herd) was performed on individual serum sample data derived from data in Swedish dairy herds. Applying the same ELISA used in the present study, for two consecutive years (1999-2000) Höglund (unpublished data) sampled 39-59 individual adult cows on 5 different farms, one sampling round taking place in spring (end of April/ start of May) and another in autumn (end of October/ November), giving 20 samplings in total. During these early analysis, results were corrected for a known positive sample on each ELISA plate and then expressed as percentage positivity. For all samplings, the PP were bootstrap sampled (1000 iterations) and a significant difference in mean PP values were defined as the 95% confidence limits (CLs) not overlapping. For all thus identified between-farm significant differences in mean PP as well as for within-year significant differences on a farm (e.g. between spring and autumn) the number of animals to be included in a sampling for the CLs not to overlap was assessed in increments of 10 animals (e.g. 10, 20, 30 and, where possible, 40 animals). The number of farms to be sampled to be more than 95% confident to detect within-year rises in antibody levels was calculated from the proportion of the total of 10 samplings resulting in a significant rise, using Monte Carlo analysis (1000 iterations) in R.

**Results**

In spring of both years, significant differences in mean antibody PP levels were identified between farms 1 and 3 (both year 1 and year 2; Table S1). Significant within-year differences were measured on all five farms, albeit not for both years on all farms (6 out of 10 occasions). In autumn, confidence intervals did not overlap for farms 1 and 2 (year 1) and farms 2 and 3 (year 2). Bootstrap analyses of numbers of animals to include found that the CLs would overlap at the 20-animal level whereas the 30-animal level was the first to identify significant differences between populations / measurements. Thus, it was concluded that sampling at least 30 animals per herd/age group would be enough to detect differences where they exist. The minimum number of farms to be sampled to be more than 95% sure to detect within-year rises in antibody levels was 4 (mean estimated number of rises detected 2.48 (95% confidence interval 1 - 4)).

Table S1 Bootstrapped mean Dictyocaulus viviparus serum antibody levels (1000 iterations) expressed as percentage positivity (PP) in five Swedish dairy herds, presented data includes all animals sampled. Farms were sampled on two occasions (spring and autumn) and for two years (1999 and 2000).

| Dairy Farm | Year | Spring^a^ PP  Mean  (95% CI) | Month sampled | Number animals sampled | Autumn^b^ PP  Mean  (95% CI) | Month sampled | Number  animals sampled |
| --- | --- | --- | --- | --- | --- | --- | --- |
| 1 | 1 | 4.5  (3.2 – 5.8) * | April | 52 | 5.6  (4.2-6.9) | December | 54 |
| 1 | 2 | 1.8  (1.3-2.3) * Ɨ | April | 59 | 4.4  (2.9-6.0) Ɨ | November | 56 |
| 2 | 1 | 3.3  (2.3-4.3) Ɨ | May | 35 | 9.1  (7.6-10.6)* Ɨ | November | 44 |
| 2 | 2 | 6.3  (1.8-10.7) | April | 44 | 2.8  (1.9-3.8)* | November | 55 |
| 3 | 1 | 2.2  (1.7-2.6)* Ɨ | May | 46 | 9.3  (7.4 -11.2) Ɨ | November | 54 |
| 3 | 2 | 5.4  (2.6 – 8.2)* | May | 45 | 7.6  (4.7-10.6) | October | 55 |
| 4 | 1 | 2.7  (1.6 – 3.6) Ɨ | April | 38 | 4.9  (3.7-6.1) Ɨ | October | 36 |
| 4 | 2 | 1.2  (0.6 – 1.9) | April | 32 | 6.0  (1.3-10.8) | September | 43 |
| 5 | 1 | 3.4  (2.0 – 4.7) Ɨ | April | 47 | 8.9  (6.5-11.2) Ɨ | December | 48 |
| 5 | 2 | 3.7  (2.3 – 5.1) Ɨ | May | 46 | 8.0  (6.2-9.7) Ɨ | December | 55 |

* significance between years

Ɨ significance within years

^a^ Spring sampling refers to sampling in April or May

^b^ Autumn sampling refers to sampling in October or November
